# Preparation of Bimodal Silver Nanoparticle Ink Based on Liquid Phase Reduction Method

**DOI:** 10.3390/nano12030560

**Published:** 2022-02-06

**Authors:** Zhiheng Yu, Tiancheng Zhang, Kaifeng Li, Fengli Huang, Chengli Tang

**Affiliations:** 1 College of Mechanical and Electrical Engineering, Jiaxing Nanhu University, Jiaxing 341000, China; yuzhiheng@jxnhu.edu.cn; 2 Zhejiang Key Laboratory of Medical Electronics and Digital Health, Jiaxing University, Jiaxing 314001, China; tcl-lily@mail.zjxu.edu.cn; 3 Faculty of Mechanical Engineering & Automation, Zhejiang Sci-Tech University, Hangzhou 310018, China; ztc950409@163.com; 4 College of Mechanical Engineering, Jiamusi University, Jiamusi 154007, China; lkfpaper@163.com

**Keywords:** liquid phase reduction, resistivity, silver nanoparticle ink, multiparticle size distribution, Horsfield packing theory

## Abstract

Improving the conductivity of metal particle inks is a hot topic of scientific research. In this paper, a method for preparing metal-filled particles was proposed. By adding filled particles to the ink, the size distribution of particles could be changed to form a bimodal distribution structure in accordance with Horsfield’s stacking model. The filling particles had small volume and good fluidity, which could fill the gaps between the particles after printing and improve its electrical conductivity without significantly changing the metal solid content in the ink. Experimental results show that the silver content of the ink slightly increased from 15 wt% to 16.5 wt% after adding filled particles. However, the conductivity of the ink was significantly improved, and after sintering, the resistivity of the ink decreased from 70.2 μΩ∙cm to 31.2 μΩ∙cm. In addition, the filling particles prepared by this method is simple and has a high material utilization rate, which could be applied to the preparation of other kinds of metal particle inks.

## 1. Introduction

Printing technology has the advantages of its simple process and environmental friendliness. In recent years, printed electronic technology has been used instead of traditional lithography technology [1]. It has become a promising manufacturing technology that can accurately print various materials on different substrates through digital control and make them electrically conductive after heat treatment [2]. At present, this technology has been widely used in the manufacturing of organic diodes, field effect transistors, radio frequency identification (RFID) tags, low-cost sensors and many other flexible electronic products [3,4,5,6]. Conductive ink [7], printing technology [8], and post-treatment process [9] determine the performance of flexible conductive patterns [10]. As one of the key factors, conductive ink directly affects the quality of flexible electronic products [11,12] and its technological development has received widespread attention.

Metallic nano-ink is the most researched and used ink due to its excellent electrical conductivity [13]. Silver nanoparticles (AgNPs) have the properties of most metal nanoparticles, such as excellent chemical stability, adsorption, antibacterial and optical properties [14,15,16]. In addition, compared with other non-metallic nanomaterials, AgNPs also have better electrical properties [17,18], which makes them increasingly important for applications in microelectronics [19], optoelectronics [20], medicine [21,22], catalysis [23,24] and biological sensing [6,25]. Thus far, AgNPs for conductive ink have been primarily synthesized by wet chemical reduction method with dispersants to prevent aggregation [26,27,28]. This method has the advantages of a short reaction time, mild reaction temperature, convenient operation and simple equipment [29]. In addition, the liquid phase reduction process is relatively mature. The desired shape and size of particles can be obtained by adjusting the factors such as additives, pH, and temperature in reduction reaction [30,31]. In order to commercialize the inks prepared by this method, the two factors of conductivity and stability must be considered [32].

At present, the mainstream method to improve the conductivity of the ink is to increase the concentration of silver particles [33,34]. However, the increase in silver concentration will not only increase the manufacturing cost, but also affect the stability of the ink, making it prone to agglomeration and precipitation, resulting in nozzle blockage or uneven print patterns [35]. Another feasible way to relieve the contraction between conductive component content and the ink stability is to establish more conductive pathways under a low content of conductive fillers [36]. The electrical conductivity of a printed pattern can be enhanced by increasing its density. Ding et al. used the one-step polyol method to prepare silver nanoparticles with multi-particle size distribution at a large scale, and they found that samples with a wider particle size distribution had better conductivity after sintering [37]. Tang et al. prepared silver nanoparticle inks with multiple particle size distributions using the microwave one-step method. Because the size of the particles in the ink is different, smaller particles can fill in the gaps between larger particles. After measurement and analysis, the solid content of the ink is 10 wt%, and its resistivity can reach 98 μΩ∙cm after sintering [38]. Currently, researchers can only broaden the size distribution range of ink particles to improve the packing density of the printed pattern. However, according to the Horsfield packing theory [39,40,41], an ideal filling model should follow a bimodal or even trimodal distribution (particle size should be concentrated in two or three regions) [42]. By liquid phase reduction method, the particle size of the generated particles mostly follows the normal distribution and it is difficult to form an accurate and reasonable distribution model.

In this paper, a new method was proposed to improve the size distribution of ink by adding filling particles. Firstly, a silver particle ink was prepared by liquid phase reduction in the laboratory, and its particle size distribution was observed by scanning electron microscopy (SEM). After that, the ideal size of the filling particles was calculated with the guidance of the Horsfield packing theory. Silver powder prepared by domestic gas atomization with appropriate size was selected as the filling particles. The filling particles was heated and oscillated by ultrasonic in the polyvinyl pyrrolidone (PVP) solution to obtain high dispersion. The filling particles were added to the silver particle ink in appropriate proportions. The ink was printed with the equipment developed by the laboratory. The printed pattern was sintered by a temperature-controlled oven, and the change of conductivity before and after improvement was analyzed.

## 2. Materials and Methods

### 2.1. Experimental Materials and Equipment

Silver nitrate (AgNO_3_, produced by Sinopharm Chemical Reagent Co., Ltd., Shanghai, China) was used as a silver source. Polyvinyl pyrrolidone (PVP, produced by Sinopharm Chemical Reagent Co., Ltd., Shanghai, China) was used as a dispersant. Silver powder prepared by domestic gas atomization (Shanghai Buwei Applied Materials Technology Co., Ltd., Shanghai, China) were selected to prepare the filling particles. The anhydrous ethanol (C_2_H_5_OH), ethylene glycol ((CH_2_OH)_2_) and glycerin (C_3_H_8_O_3_), used in the experiment, were all from Jiangsu Qiangsheng Functional Chemical Co., Ltd., Jiangsu, China. PI film (Shenzhen Meizanda Technology Applied Materials Co., Ltd., Shenzhen, China) was chosen as the printing substrate.

USA Sonics (VC800, Sonics & MATERIALS Inc., Newtown, CT, USA) was used for the ultrasonic heating and oxidation treatment of the samples. Centrifuge (GENIUS 16K, Changsha Xinao Instrument Co., Ltd., Changsha, China) was used to centrifuge the samples. The samples were dried by a rotary evaporator (Rotavapor R100, BUCHI Ltd., Flawil, Switzerland). The ink was sintered in a temperature-controlled oven (DHG-9055A, Shanghai Bluepard Experimental Instrument Co., Ltd., Shanghai, China).

### 2.2. Preparation of Silver Nanoparticle Ink

Conductive ink prepared in the laboratory was used in this experiment. We added 0.68 g of PVP and 0.34 g of silver nitrate to 40 mL of ethylene glycol. After magnetic stirring for 15 min, the solution was stirred by ultrasound. Ultrasonic power output was set to 85% (680 W), and the ultrasonic stirring time was 15 min. The purification of the silver nanoparticles was conducted by centrifugation. They were separated from the PVP and ethylene glycol by centrifugation at 9000 rpm for 15 min. The obtained precipitates were then dissolved in ethanol and were centrifuged three times in succession. The mixture was dried by rotary evaporator. Ethanol, ethylene glycol and glycerol were added as solvents to the dried sample, and the weight ratio of the drops is 10:9:1. A silver nanoparticle conductive ink with a weight ratio of 15 wt% was prepared.

### 2.3. Preparation of Silver-Filling Nanoparticles

In this experiment, uniform spherical silver powder was used to prepare the filled particles. Firstly, 0.6 g PVP was added to 40 mL deionized water and stirred until it was completely dissolved. Secondly, adding the 0.17 g commercial silver powder into the solution, and the mixture was magnetically stirred until the silver powder settled to the bottom of the solution. Then, the mixture was heated and stirred by ultrasonic for 15 min. After completion of ultrasound heating, the mixture was centrifuged at 9000 rpm for 15 min by centrifugation, then the supernatant was removed and ethanol was added to centrifuge again. The obtained mixture was poured into Petri dishes and dried at room temperature to form the silver nanoparticle powders.

### 2.4. Printing and Sintering

**Figure 1:**
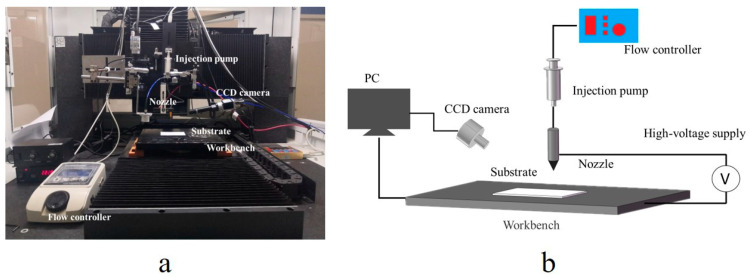
The printing equipment developed by the laboratory: (**a**) experimental equipment; and (**b**) equipment schematic diagram.

The ink was printed using the equipment displayed in Figure 1a, developed by the laboratory. A printing module is installed on the three-axis moving platform. The printing module includes a nozzle, an injection pump, an injection hose and an injection pump controller (TH-2A, Longer Precision Pump Co., Ltd., Baoding, China). The printing nozzle was a stainless-steel nozzle with an inner diameter of 0.33 mm and an outer diameter of 0.51 mm. When printing, the nozzle height was set to 0.5 mm, the flow rate was set to 0.2 mL/h, the printing speed was set to 40 mm/s and the length of the print line was set to 30 mm. By controlling the three-axis motion platform, the relative motion between the workbench and the nozzle was achieved and the conductive pattern was printed. After printing, the printed pattern was sintered by a temperature-controlled oven. PI film was selected as the printing substrate and the sintering time was set to 20 min. The schematic diagram of the printing equipment is shown in Figure 1b.

### 2.5. Characterizations

The solid content of ink was measured by a synchronous thermal analyzer (SDT-650, TA Instruments Co., Ltd., New Castle, DE, USA). The surface morphologies of the written pattern before and after sintering were observed by scanning electron microscopy (S-4800 FESEM, Hitachi Ltd., Tokyo, Japan). A multimeter (VC9801A+, Shanghai Shengheng Instrument Co., Ltd., Shanghai, China) was used to test the resistance value of the sintered pattern. A Step meter (DektakXT, Boyue Instruments Co., Ltd., Shanghai, China) was used to measure the thickness and width of the sintered pattern. The resistivity was calculated from these data.

## 3. Results and Discussion

### 3.1. Effect of Silver Particle Concentration on Dispersion

**Figure 2:**
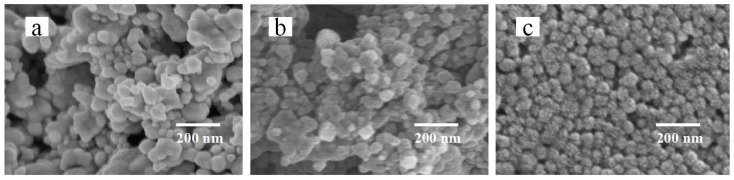
SEM image of filled particles: (**a**) SEM image with silver particle weight ratio of 1.64 wt%; (**b**) SEM image with silver particle weight ratio of 0.82 wt%; and (**c**) SEM image with silver particle weight ratio of 0.41 wt%.

Silver powder prepared by gas atomization has the advantages of uniform morphology and concentrated particle size distribution [43] and is an ideal choice for filling particles. However, because the particles prepared by this method cannot be dispersed in organic solvent, corresponding chemical treatment is needed to make them have certain dispensability. As a synthetic water-soluble polymer, PVP can be soluble in both water and most organic solvents with low toxicity, making it an ideal dispersant material [44]. The silver nanoparticles wrapped by PVP cannot only be evenly dispersed in the organic solution, but are also not easily agglomerated, which is beneficial to improve the stability of printing ink. At the same time, due to the poor conductivity of PVP [45], the excessive content of PVP will affect the conductivity of printed patterns after sintering. Therefore, it is necessary to determine the optimal ratio of PVP to silver nanoparticles. In this experiment, a uniform spherical silver particle with a particle size of 50 nm was selected, 0.6 g PVP was dissolved in 40 mL pure water and stirred until it was completely dissolved. The silver powder was added to form three groups with a silver particle weight ratio of 1.64 wt%, 0.82 wt% and 0.41 wt%, respectively. After ultrasonic stirring and centrifugation, scanning electron microscopy (SEM) was used to observe the surface morphology of each group of samples. The result is shown in Figure 2. It can be found that the mixture of 1.64 wt% shown in Figure 2a and 0.82 wt% shown in Figure 2b are agglomerated. The silver particles of 0.41 wt% shown in Figure 2c are evenly dispersed. This may be due to the high concentration of silver particles, the PVP packaging not being uniform and easy agglomeration during stirring. Considering the conductivity of silver particle inks, the optimal weight ratio of silver particles is determined to be 0.41 wt%.

### 3.2. Effect of Silver Particle Size on Dispersion

**Figure 3:**
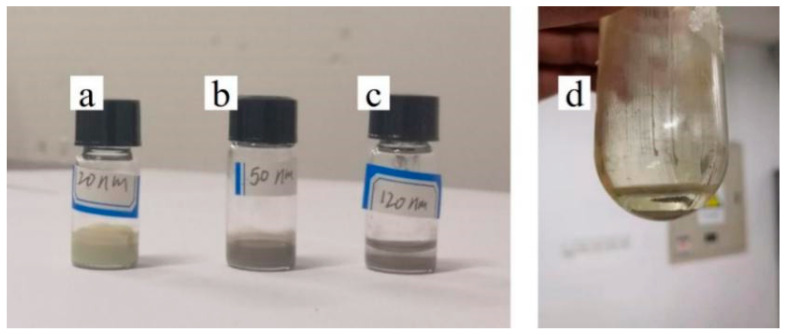
Image of the filling particles after adding ethanol and leaving for 2 h: (**a**) the 20 nm filled particles were prepared according to the weight ratio of 0.41 wt%; (**b**) the 50 nm filled particles were prepared according to the weight ratio of 0.41 wt%; (**c**) the 120 nm filled particles were prepared according to the weight ratio of 0.41 wt%; (**d**) the 120 nm filled particles were prepared according to the weight ratio of 0.2 wt%.

To explore the size range of the filling particles that can be prepared, the silver nanoparticles with sizes of 20 nm, 50 nm and 120 nm were purchased for this experiment. All particles were prepared by Shanghai Buwei Applied Materials Technology Company through gas atomization. We dissolved 0.6 g PVP in 40 mL pure water and stirred it until it was completely dissolved. Silver particle was added to make its weight ratio reach 0.41 wt%. After ultrasonic synthesis, centrifugal purification and drying, the silver granule powder which can be used as filling particle was obtained. To analyze the dispersion of silver particles, the prepared silver particle was added into anhydrous ethanol. Figure 3 shows the phenomenon of each sample after standing for 2 h. The inks with silver-filled particles of 20 nm shown in Figure 3a and 50 nm shown in Figure 3b presented uniform color and no precipitation at the bottom, while the inks with silver-filled particles of 120 nm shown in Figure 3c presented obvious stratification. To further analyze the feasibility of preparing large sized filled particles, we reduced the weight ratio of silver particles to 0.2 wt% when preparing the filled particles. As shown in Figure 3d, the prepared particles were still unable to be effectively suspended in the organic solvent, so stratification occurred after only 20 min. This may be due to the larger size of the 120 nm silver particle (13 times larger than the 50 nm particle), meaning it is also heavier. This results in 120 nm particles settling out of the liquid more easily than other small particles. Therefore, this method can only be applied to the preparation of silver-filled particles with a particle size below 50 nm.

### 3.3. Effect of Filling Particles on the Conductivity of Printed Patterns

**Figure 4:**
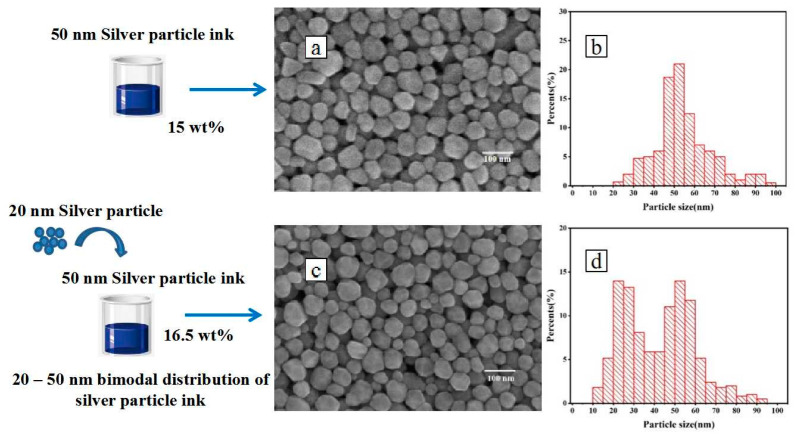
Schematic diagram of the preparation of the bimodal silver particle ink: (**a**) SEM image of the silver particle ink prepared by liquid-phase reduction method; (**b**) particle size distribution diagram of silver particle ink prepared by liquid-phase reduction method; (**c**) SEM image of silver particle ink after adding filling particles; and (**d**) particle size distribution diagram of silver particle ink after adding filling particles.

To analyze the effect of the filling particles on the conductivity of the printed pattern, nano-ink was prepared as shown in Figure 4, with a bimodal distribution of silver nanoparticles. Firstly, a silver nano-ink with a silver solid content of 15 wt% was prepared by the liquid-phase reduction method under laboratory conditions. Secondly, the surface morphology was analyzed by scanning electron microscope (SEM) shown in Figure 4a, to determine the average particle size was 50 nm (first-order particles) and the size distribution was presented in Figure 4b. Based on the theory of the Horsfield packing model, the size of the second-order particles used for filling should be 20.7 nm, and the number ratio of the first-order particles should be 1:1. In addition, the mass of the second-order filled silver nanoparticles could be calculated as follows:

*m*_2_ = (0.414^3^ × *m*_1_ × *ρ*_2_)/*ρ*_1_ (1)

where *m*_1_ is the mass of first-order silver nanoparticles, which can be calculated by multiplying the mass of silver ink and its weight ratio; *ρ*_1_ is the density of first-order particles; and *ρ*_2_ is the density of second-order particles. Since the particles used in this experiment were all silver nanoparticles, *ρ*_1_ and *ρ*_2_ are relatively the same.

**Figure 5:**
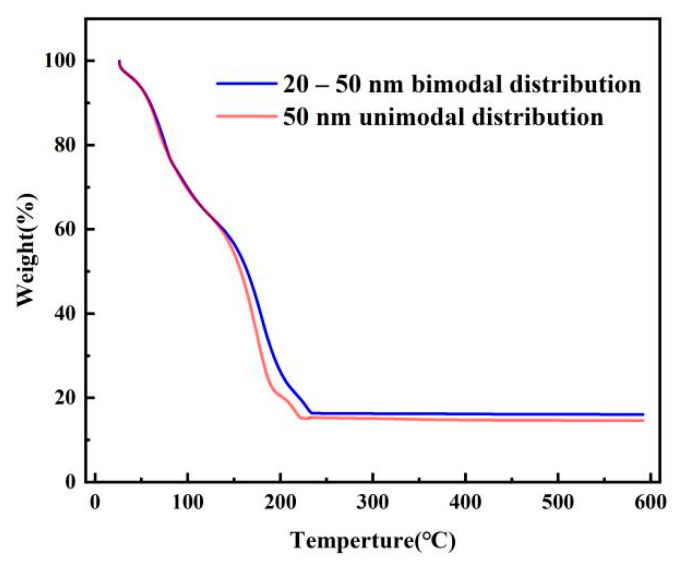
Thermal weight loss of silver nanoparticle ink prepared by liquid-phase reduction method before and after adding filling particles.

According to previous experimental results, commercial spherical silver powder with particle size of 20 nm was added into 40 mL PVP solution to form a mixture with silver weight ratio of 0.41 wt%. After ultrasonic stirring and purification, the filled particles with a particle size of 20 nm were prepared. The silver nanoparticle ink prepared by the liquid phase reduction method was divided into two parts, and one part was added with 20 nm uniform spherical silver filled particle, while the other part was used as the reference group. The SEM image of the ink after adding filling particles was shown in Figure 4c, and it can be found that the silver filling particles with a particle size of 20 nm were uniformly distributed, and that the particle size approximately obeyed the bimodal distribution demonstrated in Figure 4d. Figure 5 shows the change in the weight ratio of silver particles before and after adding filling particles measured by the TGA instrument. The weight ratio of the ink only increased by 1.53 percentage points after adding silver particles. This is the reason for which the volume of the filling particles was much smaller than the first-order silver nanoparticles. Therefore, this method can significantly improve the particle size distribution without significantly increasing the weight ratio of the ink.

**Figure 6:**
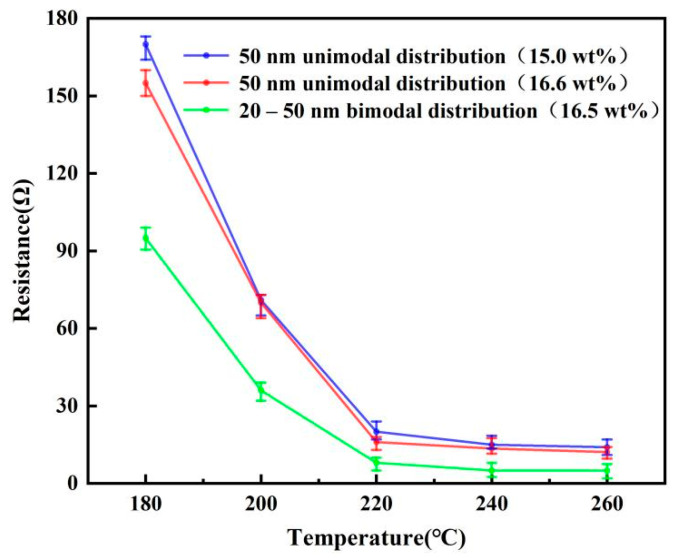
The relationship between the sintering temperature and the resistance of the different ink.

**Figure 7:**
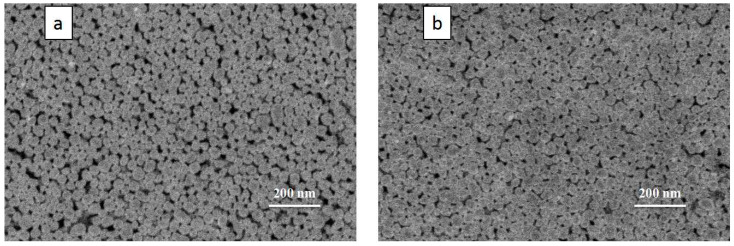
SEM images of the silver nano-ink after sintering: (**a**) SEM image of the sintered pattern of the silver nano-ink without filling particles (16.6 wt%); and (**b**) SEM image of sintered pattern of the silver nano-ink with filling particles (16.5 wt%).

In order to compare the conductivity of the ink with and without the filling particles, the ink was printed with the equipment developed by the laboratory and sintered by a temperature-controlled furnace. Considering the fact that the solid content of ink will increase after adding filled particles, and the change of solid content of ink may affect the electrical conductivity. An additional silver nanoparticle ink was prepared by liquid phase reduction method for comparison. Its average particle size was 50 nm, and it had a similar solid content (16.6 wt%) to the ink with filled particles. To seek the best sintering parameters, multiple sets of patterns were printed with three kinds of inks, and they were sintered at 180, 200, 220, 240 and 260 °C for 20 min, respectively, the sintering results are shown in Figure 6. With the increase in sintering temperature, the resistance value of the printed pattern gradually decreased and tended to be stable. When the sintering temperature is 260 °C, the resistance values of 15.0 wt% unimodal, 16.6 wt% unimodal and 16.5 wt% bimodal inks reach the lowest point, which is 14.2 Ω, 12.4 Ω and 5.1 Ω, respectively. The resistance variation trend of the 50 nm unimodal distribution inks with solid content of 15 wt% and 16.6 wt% was similar, and the slight increase in solid content did not significantly improve the electrical conductivity of the printed pattern. However, after sintering, the resistance value of ink with 20–50 nm bimodal distribution was much lower than that of ink without filling particles. To further explore the reason, the surface morphology of the conductive pattern after sintering was observed through the SEM image and the results were shown in Figure 7. Compared with the conductive pattern shown in Figure 7a printed by the original silver nano-ink, the conductive pattern printed by the silver nano-ink filled with particles shown in Figure 7b was better, which had a denser surface and a smaller porosity after sintering. Because the filling particles had a small volume and good fluidity, they could fill the gap between 50 nm particles and increased the number of conductive paths inside the pattern, thus improving the density of the printed pattern. The results show that adding filling particles can effectively improve the internal structure of the printed pattern after sintering. The resistivity of the improved printed pattern was 31.2 μΩ∙cm after measurement. Additionally, compared with the printed pattern before the improvement, the resistivity of 70.2 μΩ∙cm was greatly improved.

## 4. Conclusions

The method was proposed to prepare high dispersibility silver nano-ink filled particles in this work. When the weight ratio of silver particles is lower than 0.41 wt%, highly dispersible silver nano-filled particles with an average size of less than 50 nm can be prepared. The experiments showed that adding filling particles can effectively improve the particle size distribution of the ink without significantly changing its weight ratio. Thereby, it can be made into bimodal nano-ink that conforms to the Horsfield stacking model. The increase in the packing density of the printed pattern can reduce its internal porosity and increase the number of effective conductive paths. As a result, the resistivity of the printed pattern decreases from 70.2 μΩ∙cm to 31.2 μΩ∙cm. In addition, the filling particles have the advantages of a simple manufacturing process, low-cost and high material utilization rate. It can be applied to improve most metal particle inks, which is of great significance to promote the development of flexible electronic technology.

## Data Availability

The data that support the findings of this study have not been made available but can be obtained from the author upon request.
